# The changing nature of work – Job strain, job support and sickness absence among care workers and in other occupations in Sweden 1991–2013

**DOI:** 10.1016/j.ssmph.2021.100893

**Published:** 2021-08-11

**Authors:** Gunnar Aronsson, Staffan Marklund, Constanze Leineweber, Magnus Helgesson

**Affiliations:** a Department of Psychology, Stockholm University, Stockholm, Sweden; b Division of Insurance Medicine, Department of Clinical Neuroscience, Karolinska Institutet, SE-171 77, Stockholm, Sweden

**Keywords:** Work environment, Iso-strain, Job demands, Job control, Social support, Sickness absence, Population study, Business cycles, Psychosocial exposure, Care workers, Nurses

## Abstract

This study examined exposure changes in three psychosocial dimensions – job demands, job control, and social support – and the associations between these dimensions and sickness absence throughout the period 1991–2013. The analyses covered periods of economic ups and downs in Sweden and periods involving major fluctuations in sickness absence. Data on care workers (n = 16,179) and a comparison group of employees in other occupations (n = 82,070) were derived from the biennial Swedish Work Environment Survey and linked to register data on sickness absence. Eight exposure profiles, based on combinations of demands, control, and support, were formed. The proportion of individuals with work profiles involving high demands doubled among care workers (14%–29%) while increasing modestly in the comparison group (17%–21%) 1991–2013. The work profile that isolated high-strain (iso-strain), i.e., high demands, low control, and low social support, was more prevalent among care workers, from 4% in 1991 to 11% in 2013. Individuals with work profiles involving high-demand jobs had the highest number of days on sickness absence during the study period and those with the iso-strain work profile had the highest increase in sickness absence, from 15 days per year during 1993–1994, to 42 days during 2000–2002. Employees with a passive work profile (low job demands and low job control) had the lowest rate and the lowest increase in sickness absence. Individuals with active work profiles, where high demands are supposed to be balanced by high job control, had a rather high increase in sickness days around 2000. A conclusion is that there is a long-term trend towards jobs with high demands. This trend is stronger among care workers than among other occupations. These levels of job demands seem to be at such a level that it is difficult to compensate for with higher job control and social support.

## Highlights

• The study covers a unique long period (1991–2013) with work exposure and sick leave.
• The iso-strain profile increased more among care workers than in other occupations.
• During a recession period, sick leave increased considerably more in high demand jobs.
• It seems difficult to compensate high job demands with job control and social support.
• The trend towards an increase in high demand jobs is most marked among care workers.

## 1 Introduction

This study examines changes in stress- and health-related psychosocial exposures and sickness absence during more than 20 years in Swedish working life. The focus is on two women-dominated large occupational groups within the Swedish health care sector, i.e. nurses and care assistants. A comparison group, including all other occupations of the Swedish working population, is also used.

Sickness absence has fluctuated a lot in Sweden during the last three decades and has increased among nurses and care assistants during this time (Marklund et al., 2019). Around the turn of the millennium, the sickness absence rate had a peak with very large costs leading to production losses in companies, and public costs for sickness absence compensation to individuals. Therefore, studies that can increase the understanding of the reasons behind increasing sickness absence rates are highly warranted. Sickness absence reflects the health of the working population but the relationship between illness and sickness absence is variable. Research has identified several factors that influence the association such as sickness presenteeism (Aronsson et al. 2000, 2021), use of sickness absence as an illness prevention strategy (Kristensen, 1991), job insecurity (Ferrie et al., 2001; Virtanen et al., 2003) and different thresholds for sickness absence between occupations (Andersen et al., 2012). The role of sickness absence compensation levels may also depend on which time perspective is chosen (Sjöberg, 2017).

This study primarily has a micro perspective. The empirical analysis is based on the job demand-control-social support model (Johnson et al., 1989; Karasek, 1979; Karasek & Theorell, 1990). This model and dimensions have been shown to have a high explanatory value for studies of the link between work environment conditions and various individual ill-health symptoms. In the current study, we described proportional changes in these three dimensions in the working population, and the association between these dimensions and sickness absence.

The sharp economic fluctuations and rapid technological development that took place during the long study period motivate that the results are related to meso- and macro-changes. However, on those levels, we lack individual and aggregated data in order to perform correlation analyses, so assumptions about associations must rely on ecological observations that can hopefully serve as a basis for further research. Nor have we been able to identify studies based on the job-demands-control-support model that have taken meso and macro contextual variations and changes into account, such as economic turbulence, level of unemployment, a budget crisis in the public sector, etc., i.e. conditions that have been shown to correlate with sickness absence in aggregated measures (Thorsen et al., 2015).

### 1.1 The job demands, control, social support model

According to the job-demands-control-support model, a combination of high job demands and a low degree of influence (low job control) at work (high-strain job) would be associated with the highest risk for ill-health. The combination of high-strain and low social support (isolated) at work has been referred to as ‘iso-strain job’ and has been in focus in most studies. Social support from work colleagues or managers is expected to be health-promoting in several ways, while a lack of social support has emerged as a risk factor for severe ill-health outcomes. Meta-analyses have provided evidence for the associations between the three dimensions and ill-health outcomes such as the increased risk for depressive symptoms (Theorell et al., 2015), heart disease (Theorell et al., 2016), symptoms of burnout (Aronsson et al., 2017), disturbed sleep (Linton et al., 2015) and back pain (SBU, 2014). The relationships are consistent and robust, but the mechanisms that explain them are less studied (Kivimäki et al., 2018).

To the best of our knowledge, no systematic reviews or meta-analyses have been conducted on the job-demand-control-support model in relation to sickness absence. A handful of prospective studies have been conducted and the results are not entirely consistent: some found significant associations while others did not (Mutambudzi et al., 2019). In a large ten-year prospective study of the German working population, Mutambudzi and colleagues found that passive (low job demands and low job control) and high-strain jobs, both of which have low levels of control, were associated with an increased risk for long-term sickness absence (Mutambudzi et al., 2019). In another one-year prospective study from Norway, only high-strain work was found to be related to increased sickness absence (Wang et al., 2014). In a large two-year prospective study on a Finnish population, the association between high-strain work and sickness absence was significant only among workers with a high socioeconomic position (Virtanen et al., 2007).

Profiles other than the high-strain profile have also been found to be related to sickness absence. A Swedish study found that being in an active job (high job demands and high job control) was related to a significantly increased risk of increased long-term sickness absence only among women (Lidwall & Marklund, 2006). In the abovementioned study (Mutambudzi et al., 2019), the researchers found an increased risk of long-term sickness absence among individuals who had a passive job. Other studies have found passive work to be related to increased sickness absence only among men (Lund et al., 2005; Suominen et al., 2007).

In summary, there is evidence that the job-demand-control-social support model has a good explanatory value for different types of ill-health. Knowledge about the association with sickness absence has not been systematically investigated and there is inconsistency in the results. There is a need for more knowledge about the influence of macro and meso-contextual changes on the three dimensions and the combinations of these.

### 1.2 Macro and meso changes during the study period

Macro and meso aspects with presumed relevance for workplace conditions and sickness absence during the period 1991–2013 can briefly be described as follows. In Sweden, as in other industrialised countries, work environment and working life underwent major structural changes, digitalization, and changed work organisations and control systems. Employees in industrial companies, with physically demanding work environments, have diminished, while the proportion of employees working in modern office work environments has grown. Also, the public sector in Sweden has changed considerably, with major rationalization programmes that have changed the nature of work and the psychosocial demands (Marklund et al., 2019).

In 2003 the Swedish Welfare Commission presented a balance sheet for developments regarding welfare throughout the 1990s. Their conclusion was that: “the 1990s can be described as a decade of mass unemployment in Sweden” (Palme et al., 2003).

The decade began with large layoffs in the industrial sector and total unemployment rose steeply from around 2 per cent in 1991 to around 8 per cent in 1993, when unemployment levelled out. In 1997 unemployment began to decline, and in 2001 it stabilised at around 4 per cent. The economic downturn in the private sector caused a delayed budget crisis in the public sector, which resulted in large staff cuts in the second half of the 1990s, mainly focused on reductions among assistant staff in the healthcare sector (Eliason, 2011, 2014).

The two primary occupational groups in the present study, nurses, and care assistants overlap to a large extent with auxiliary nurses and assistant nurses, who according to available statistics were highly influenced by the staff cuts in the 90s. The number of auxiliary nurses was reduced by 38.7 percent and assistant nurses by 33.6 per cent (Eliason, 2011, 2014). During the same period, the reduction among all nursing positions (our second study group) was 5 per cent (Swedish Association of Local Authorities and Regions, 2002).

Economic recovery and decreasing sickness absence characterised the years before the international financial crisis in 2008. However, the public sector in Sweden was rather unaffected by that crisis and there was no such large budget deficit as in the 90s. In 2010 an upturn in the business cycle began and the last years of our study period were stable in terms of macro aspects.

Business cycles and organisational instability can be presumed to influence and change job demands, control, and social support at workplace level. Kivimäki et al., for example, found that an increase in sickness absence during organisational downsizing was partially explained by accompanying increases in physical demands and job insecurity and a reduction in job control (Kivimaki et al., 2000) and the studies of Noblet and colleagues on New Public Management models (Noblet et al., 2006; Noblet & Rodwell, 2009) showed that increased job control and social support were useful ways of overcoming New Public Management induced work intensification, increased influence of external stakeholders and increased use of performance monitoring.

### 1.3 Changed diagnoses and sickness levels among men and women

The macro and meso changes have been accompanied by a continuous shift in the composition of a medical diagnostic pattern of compensated sickness absence. Statistics regarding cases of sickness absence from the Swedish Social Insurance Agency show that the proportion of individuals with ongoing sickness absence due to psychiatric diagnoses (International Classification of Diseases, version 10 (ICD-10): F00–F99) has increased from 29.6 per cent in 2005 to 48.1 per cent in 2019 (2005 was the first year when sickness absence diagnoses were available). The corresponding figures for musculoskeletal diagnoses (ICD-10: M00-M99) decreased from 29.6 to 16.6 per cent during the same period (Swedish Social Insurance Agency, 2020).

The change in diagnostic patterns is almost linear and seems only weakly related to business cycles. We presume that the changes and the linearity reflect deeper structural changes in the nature of work: from a working life with a high proportion of workers exposed to physical load to a working life with more exposure to mental and cognitive load.

Another stable and long-term trend is that women have increased their share of the total sickness absences, and in 2020, women's share of the sickness absences was approximately 67 percent. In accordance with two relatively recent reviews on gender and sickness absence in Sweden, the focus in the current study is on psychosocial work environment exposure structures, not on individual or biological aspects. The conclusion from both reviews is that the higher sickness absence rates among women are mainly a structural problem, as workplaces with a majority of female employees seem to be worse regarding exposure to work environment factors such as high job demands and low control, that are associated with health risks and increased sickness absence rates (FORTE, 2015; Sverke et al., 2017).

### 1.4 Aims

The study has three aims. The first aim was to describe exposure changes in the eight combinations of three psychosocial dimensions – job demands, job control, and social support over the years 1991–2013 in nurses and care assistants and a comparison group with the rest of the Swedish working population.

The second aim was to describe and analyse the relationships between these eight exposure profiles and individually compensated sickness absence over the three years following participation in the survey under periods of increasing as well as decreasing sickness absence and under a shifting macro and meso context.

A third and explorative aim was to contribute to a better understanding of how contextual factors such as business cycles and technological and organisational development changes are mediated to changes in psychosocial exposure at workplace level. For this aim, we have relied on ecological observations and associations.

## 2 Materials and methods

### 2.1 Study population

The study population consisted of employed men and women, between the ages of 16 and 64, who participated in the Swedish Work Environment Survey (SWES) between 1991 and 2013 (Table 1). The distribution of participants in SWES (1991–2013), according to the Standard for Swedish Occupational Classification from 1996 (SSYK-96), was: 1,843 nurses (SSYK-96: 323); 1,210 specialist nurses (SSYK-96: 223), and 13,126 care assistants and other social workers (SSYK-96: 513). These groups will hereafter be referred to as care workers. Further, a comparison group, that comprised individuals from all other occupations (n = 82,070) was used.

**Table 1:** Characteristics of the study population stratified on Care workers and All other occupations (n = 98,249).

	Care workers	All other occupations
	N (%)	N (%)
**All**	16,179	82,070
**Sex**		
Men	1,363 (8.4)	45,925 (56.0)
Women	14,811 (91.6)	36,095 (44.0)
*Missing data on sex*	*5*	*50*
**Age (mean)**		
Period 1 (1991–1995)	40.9	42.4
Period 2 (1997–2001)	43.5	45.0
Period 3 (2003–2007)	44.2	44.5
Period 4 (2009–2013)	46.5	45.0
**Educational level**		
Elementary school	1,237 (7.7)	15,227 (18.6)
Upper secondary school	5,053 (31.3)	28,788 (35.1)
University	9,879 (61.1)	37,960 (46.3)
*Missing data on education*	*10*	*95*

### 2.2 The Swedish work environment survey (SWES)

The SWES has been conducted every second year since 1989. Each wave has comprised of between 8,135 and 14,300 individuals which corresponded to a response rate of between 66 and 88 per cent (Swedish Work Environment Agency, 2014). The SWES populations are representative samples of the working population in Sweden. One of its fundamental objectives is to measure how frequently a certain exposure is encountered over certain time periods, such as a common workweek. An individual reports how often he or she is exposed to something at work, such as how often he or she must skip lunch, work overtime or take work home. Examples of frequency scales from SWES include: Every day, A few days per week (1 day out of 2), One day per week (1 day out of 5), A few days per month (1 day out of 10), and Not at all over the last 3 months (an English version of the SWES is attached as a supplementary file). These types of scales were developed as alternatives to intensity scales, which are commonly used to inquire about individuals’ perceptions of their work environment. Tests have shown that inter-rater reliability is higher for frequency estimates, as the range in responses from people with the same working conditions is smaller, as well as the variation in estimates over time of the same working conditions (Wikman, 1991, 2006).

Frequency estimates are considered to provide relatively individual independent exposure data, which are thus useful for comparisons over time, for example, in terms of how many are included in a certain job demands-control-support profile. The validity and reliability of the variables used in the SWES were tested at the workplace, where the actual conditions were known and could be compared to other types of information such as administrative and technical information. Moreover, responses to different formulations of questions were used and compared (Wikman, 1991).

### 2.3 Items and indexing – job demands, job control, and social support

Data on exposures of psychosocial work environment were in this study obtained from 12 iterations of SWES, from 1991 to 2013. Similar to other studies and the balance between statistical power and exposure contrasts, the response options for the chosen items were dichotomised closest to the upper quartile to indicate adverse conditions (Helgesson et al., 2020, 2021; Leineweber et al., 2020; Marklund et al., 2019).

*Job demands* were calculated based on the responses to the following four items:

* ‘Do you have so much work that you must miss lunch, work late, or take work home?’ (Exposed: Half of the working time, the entire working time)
* ‘Is your work so stressful that you do not have time to talk or even think about something other than work?’ (Exposed: 3/4 of the working time – almost the entire working time)
* ‘Does the work require your full attention and concentration?’ (Exposed: Almost the entire working time)
* ‘Far too much/little to do at work?’ (Exposed: Far too much to do at work).

*Job control* was calculated based on the responses to the following four items:

* ‘Do you participate in decisions on the arrangement of your work?’ (Exposed: Most of the working time to not at all)
* ‘Are you able to determine when various work duties are to be carried out?’ (Exposed: No, not at all)
* ‘Do you have the opportunity to determine your work pace?’ (Exposed: 1/10 of the working time, No, not at all)
* ‘Too little/much influence?’ at work (Exposed: Little or too little influence at work)

Individuals were considered exposed to *job demands* and to *job control* if their responses to two of four items, respectively, indicated exposure.

An index for *social* support was created based on the two following questions:

*‘Are you able to get support and encouragement from supervisors when work feels difficult?’
*‘Are you able to get support and encouragement from colleagues when work feels difficult?’

Individuals were considered to have low *soci*al support when their responses indicated that they never received support from either their supervisors or their colleagues when the work felt difficult. Individuals who responded that they mostly not, mostly, or always got support from either supervisors or colleagues were considered to have high *socia*l support.

The following eight exposure profiles, based on the abovementioned items, were used in the analyses:

1. Low strain collective = low *job demands*, high *job control*, high *social support*
2. Low strain isolated = low *job demands*, high *job control*, low *social support*
3. Passive collective = low *job demands*, low *job control*, high *social support*
4. Passive isolated = low *job demands*, low *job control*, low *social support*
5. Active collective = high *job demands*, high *job control*, high *social support*
6. Active isolated = high *job demands*, high *job control*, low *social support*
7. High-strain collective = high *job demands*, low *job control*, high *social support*
8. High-strain isolated (iso-strain) = high *job demands*, low *job control*, low *social support*

### 2.4 Register data

Data on sickness absence, age, and educational level were obtained for all participants in SWES from the Longitudinal Integrated Database for Health Insurance and Labour Market Studies (LISA) hosted by Statistics Sweden. Data on all these variables were available from the year 1993.

#### 2.4.1 Age and educational level

Age was categorised into the following categories: <35 years, 35–44 years, 45–54 years, and >54 years. Educational level was categorised into elementary school (<10 years of education), upper secondary school (10–12 years of education), university (>12 years of education).

#### 2.4.2 Sickness absence

Net days of sickness absence were used in the analyses. Here, for example, two days of half-time sickness absence or four days on ¼ sickness absence equaled one net day. In Sweden, the first day of sickness absence is a qualifying day not compensated for and the following 13 days are covered by the employer. From day 15 compensation for sickness absence is covered by the Social Insurance Agency. Since register data only include sickness absence spells compensated for by the Social Insurance Agency, only sickness spells exceeding 14 days were included. The variable sickness absence used does not include periods of looking after children or periods when the ability to work is reduced through pregnancy. In addition, all cases of sickness absence in this study were certified by a physician, as this is a requirement for periods lasting more than 7 days.

Sickness absence was calculated as the average sum of sickness absence days over the three years following the interview in the SWES. For those interviewed in the SWES during year 1993, the average days of sickness absence were measured during the years 1994–1996. Those who were interviewed in year 1995 were linked to sickness absence during the years 1996–1998 and so on. For those interviewed in the 1991 version of SWES, sickness absence data were, however, only available for the years1993 and 1994.

### 2.5 Statistical processing

The results were organised as follows: 1) a descriptive part and 2) an analytic part. The descriptive part was divided into two parts: A) how the proportions of individuals with each of the eight combinations of exposures (exposure profiles) changed between 1991 and 2013 and B) how the annual average of sickness absence days during the three years following the year of participating in SWES changed for each of the eight profiles based on *job demands*, *job control,* and *social* support. The descriptive part was calculated and presented without adjustment for any confounders.

In the second part, Incidence Rate Ratios (IRRs) of the annual average of sickness absence days three years after the interview in SWES were calculated. Despite the large number of participants, some profiles over a period of a few years ended up including relatively few people. To gain a more accurate understanding of the differences between the various exposure profiles, data were pooled into four periods that represented periods of either an increase or decrease in the societal rate of sickness absence. Each measurement point was therefore based on four groups including three iterations of SWES, that is: 1) 1991–1995, 2) 1997–2001, 3) 2003–2007 and 4) 2009–2013.

Corresponding data regarding sickness absence data were measured for the three years following every iteration of SWES, that is 1993–1998, 1998–2004, 2004–2010, and 2010–2016. IRRs were calculated (through a command in SPSS statistical software, *GENLIN*) from the average annual number of days on sickness absence three calendar years after the interview in SWES. Here, a generalised linear model with an error distribution other than a normal distribution was estimated. The low strain collective profile was considered as the reference group. Adjustments were made for age and educational level. Individuals with missing data for any of these variables were excluded from the analysis. Very few had, however, missing data neither on exposure variables nor on the outcome variable. As the proportion of male care workers was very low, men and women were not analysed separately. All analyses were performed using SPSS Statistical Software version 26.

### 2.6 Ethical considerations

The Regional Ethical Board in Stockholm approved this study (no. 2018/223-31/5) and waived the requirement that informed consent from the research subjects should be collected.

## 3 Results

### 3.1 Changes in exposure profile proportions, 1991–2013

**Fig. 1:**
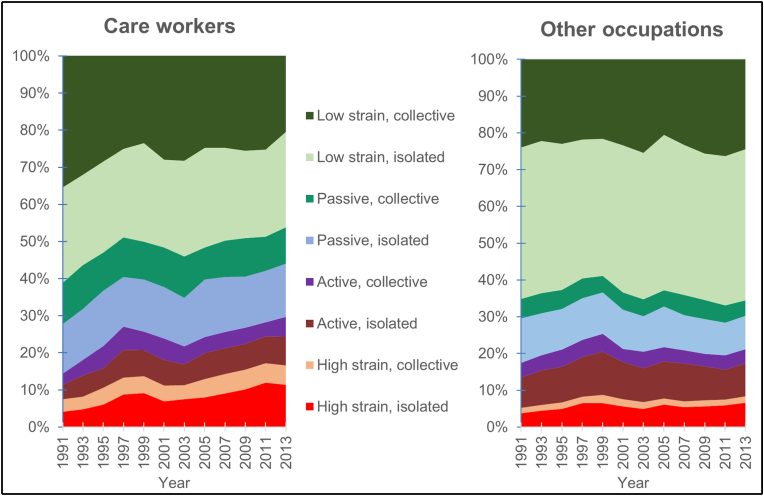
Changes in the exposure profile proportions for the eight combinations of job demands, job control, and social support between 1991 and 2013.

Fig. 1 shows the changes in the proportions of the eight exposure profiles from 1991 to 2013. As can be seen, the exposure profiles differed considerably in size and the way in which they changed during the study period. The profiles including low strain were most common in both occupational groups. At the beginning of the study period, a majority of care workers had the low strain collective profile, while the low strain isolated profile was most prevalent in the other occupations group, both at the beginning as well as at the end of the study period, applying to 41 per cent of the employees.

The profiles differed in terms of the changes they underwent over the course of the study period. Overall, there was a tendency of distinct shifts towards more job strain among care workers, while the changes regarding other occupations group were quite small.

**Fig. 2:**
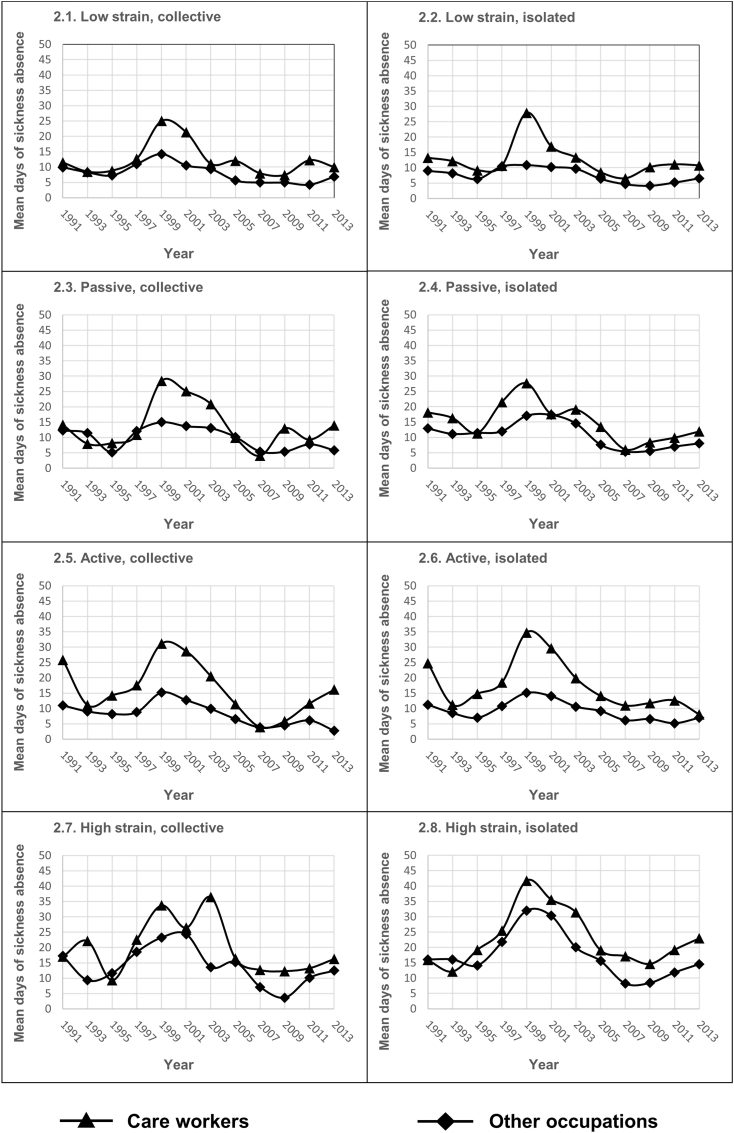
Sickness absence days for the combinations of job demands, job control, and social support. The year for exposure measurement and mean of annual days of compensated sickness absence days during the subsequent three years (for exposure 1991 the follow-up period was the mean of 2 years–1993–94).

For care workers, the low strain collective profile started at 35 per cent in 1991 and continuously decreased to 21 per cent in 2013. For the iso-strain profile, however, the percentage more than doubled over the years, going from 4 to 11 per cent. As can be seen in Fig. 2, the upturn was strong during the late 1990s when large-scale staff reductions were occurring – an increase that did not decline afterward but which continued to rise, although at a slower pace. As mentioned earlier, this is also the profile that has emerged in various studies as the most problematic from a health point of view. In the all-other occupations group, there was also an increase for the iso-strain profile, but only from 4 to 7 per cent.

Among care workers, there was an increase from 4 to 8 per cent for the active isolated profile, which can also be considered a problematic profile from a health point of view. Just as with the iso-strain profile, the main increase occurs at the end of the 1990s and the proportion does not return to the 1991 level.

Over the course of the entire study period, the four profiles characterised by high demands roughly doubled among the care occupations, from 14 per cent in 1991 to 29 per cent in 2013, while these profiles increased from 17 to 21 per cent for the all-other occupations group.

As there were few men among the care workers, statistical comparisons between the sexes could not be carried out. In a separate analysis, we compared exposure changes among women in the care occupations with that of women in the all-other occupations group. For the most unfavourable profiles, the trend was somewhat more negative among the care workers than for the all-other occupations group. The proportion of iso-strain profiles increased from 4 to 12 per cent among women in care work, while increasing from 5 to 9 per cent among women in the all-other occupations group (data not shown). The proportion of active isolated profiles doubled from 4 to 8 per cent among the female care workers and from 8 to 10 per cent among the women in all other occupations.

### 3.2 Exposure profiles and compensated sickness absence

Figs. 2.1 to 2.8 show the sickness absence levels and trends between 1991 and 2016 (interviews from 1991 to 2013) for the eight exposure profiles, calculated as the sum of the annual number of compensated sickness absence days over the three years following the year of participating in SWES. With only a few, minor exceptions during all years, the all-other occupations group had fewer sickness absence days than the care workers.

The differences in the levels between care workers and the other occupations were small in the sample of 1991 (sickness absence 1993 and 1994) except for the active profiles (2.5 and 2.6). In the exposure measurement of 1993, the care worker profiles were rather similar in 1994-96 with one exception. When staff reductions and downsizing started and were prevalent in the second part of the 90s, a clear gap in absence levels between care workers and other occupations soon became evident. After about ten years, in 2007 after the rapid increase in sickness absence, all subgroups were back at almost the same level of sickness absence as before.

Among the care workers, those with the iso-strain profile (Fig. 2.8) started at 15.9 days of registered sickness absence in 1993-94 and increased to 41.8 days in the sample of 1999 and sickness absence data from 2000 to 2002. In 2010–2012, the iso-strain profile was back on 14.6 days before a new increase to 23.0 days 2014–2016. In the all-other occupations group, the pattern among those with the iso-strain profile was similar to that of the care workers, although the increase was considerably less.

Regarding the high-strain collective profile (Fig. 2.7), the number of sickness absence days varied somewhat irregularly for the care workers throughout the study period, which is probably related to the low number of respondents in this profile. In general, for the passive collective and isolated profiles (Figs. 2.3 and 2.4), the increase in sickness absence days was less pronounced than for the active profiles (Figs. 2.5 and 2.6). Employees with active profiles showed a marked increase in sickness absence around the turn of the century (1997–2001) and the time for returning to lower levels was longer than in the passive profiles. Seen over time, the passive isolated profile groups showed the lowest levels of sickness absence. In the all-other occupations group, the increase in sickness absence for the corresponding passive and active profiles was considerably weaker (Figs. 2.3–2.6).

The increase in sickness absence days among the low strain collective and low strain isolated profile groups (Figs. 2.1 and 2.2) in the late 1990s was more pronounced among care workers than among the corresponding profiles in the all-other occupations group. Care workers in the low strain profiles had less than 10 days of sickness absence in the sample of 1995. In the 1999 sample, the level was between 25 and 28 days. Thereafter, the sickness absence among care workers in the two low strain profiles decreased and was close to the number of sickness absence days for the all-other occupations group around 2008–2010. After 2010 there was a slight increase mainly for the care workers. In the same two profiles (Figs. 2.1 and 2.2) in the all-other occupation group, there was hardly any change at all in the number of sick days at the turn of the millennium.

An overall comparison of the care workers indicates the importance of job demands. If profiles with low and high job demands but equal in control and social support are compared (four possible comparisons), the tendency is clear. During the turbulent years 1998–2002, the high-demand profiles had between 5 and 10 more sickness absence days per year than the low-demand profiles. This tendency was present in the entire material, but from 2008 onwards it was not so accentuated. Similarly, the trend was the same among other occupations but weaker.

### 3.3 Sickness absence among care workers during different periods

As mentioned, despite the large number of participants, some profiles in some years ended up including relatively few people. For statistical reasons, we pooled the data into four periods so that each measurement point was based on three measurements. The periods can shortly be described as follows concerning contextual aspects (Eliason, 2011, 2014; Palme et al., 2003; Swedish Social Insurance Agency, 2014). During period 1 (interviews 1991–1995) there was a strong increase in unemployment in Sweden mainly in the private work sector, while care workers were rather unaffected. However, a strong reduction of assisting health personnel started at the end of period 1 and continued during period 2 (interviews 1997–2001) (Eliason, 2011, 2014). Period 3 (interviews 2003–2007) was characterised by economic recovery and decreasing sickness absence. For those interviewed in 2007, their measurement of sickness absence was from the years of 2008–2010 when the international financial crisis occurred. However, the public sector in Sweden was rather unaffected by the crisis and there was no such large budget deficit as in the 1990s. There were also changes in the regulations for sickness insurance in July 2008, where the requirements for receiving sickness absence became tightened. Sickness absence was at its lowest level around 2009–2010. In 2010, two years after the financial crisis started an upturn in the business cycle began. The fourth period (interviews 2009–2013) was stable in terms of macro aspects.

As a complement to Fig. 2, Table 2 shows the proportion of care workers and other workers with compensated sickness absence more than 14 days during the four different periods. As can be seen, the share of care workers was considerably higher than among other workers. If we look at the care worker profiles, the proportion who were absent due to sickness shows a clear increase during period 2 and then gradually goes back to levels similar to the levels during period 1.

**Table 2:** Percentage of all care workers and other occupations that during the three-year follow-up after the year of interview had at least one spell of compensated sickness absence more than 14 days during the period 1993–2016. Data for the four three-year periods 1991–2013.

		1991–1995	1997–2001	2003–2007	2009–2013
**Care workers**	Low strain, collective	27.0	38.0	29.2	29.9
	Low strain, isolated	27.0	35.0	29.1	29.4
	Passive, collective	27.7	40.5	30.5	30.3
	Passive, isolated	30.9	41.6	34.0	33.1
	Active, collective	37.1	43.2	33.9	30.8
	Active, isolated	29.0	44.4	36.4	28.6
	High-strain, collective	32.7	47.8	39.8	33.6
	High-strain, isolated	34.7	48.5	45.0	44.7
**Other occupations**	Low strain, collective	21.9	25.6	20.4	17.2
	Low strain, isolated	21.0	24.8	19.5	16.8
	Passive, collective	28.4	29.4	24.9	21.3
	Passive, isolated	27.8	31.4	24.4	21.7
	Active, collective	22.5	27.0	21.5	16.6
	Active, isolated	21.6	26.8	21.5	18.4
	High-strain, collective	28.8	40.9	28.3	28.0
	High-strain, isolated	30.6	41.0	31.3	28.1

The development in the profiles high-strain, both collective and isolated, is remarkable. Despite the fact that the proportion who were absent due to sickness in these profiles was already high in period 1, there was a very sharp increase during period 2 with a slower return than other profiles. This is most noticeable in the profile high-strain isolated, which increased from an already high 34.7 percent during period 1 to 48.5 per cent during period 2 and then remained at high values even during periods 3 and 4 (45.0 and 44.7 respectively). There was a similar tendency in these profiles also among other occupations but on a much lower level.

The Incidence Rate Ratios (IRRs) of sickness absence among the care workers were calculated for the exposure profiles, using the low strain collective profile as the reference. The results based on the crude and adjusted model (Table 3) largely confirmed the results presented in Fig. 2 concerning the importance of job demands. In the adjusted model, all 4 high-demand profiles had statistically significant elevated levels during all four periods with the exception of the active collective profile during the fourth period.

**Table 3:** Incidence rate ratios (IRRs) with 95% confidence intervals (CIs) for days of compensated sickness absence among care workers in different exposure profiles compared to the low strain collective profile. Four periods.

			Crude Model	Adjusted model^a^
		n (%)	IRR (95% CI)	IRR (95% CI)
**Period 1**	Low strain, collective	1280 (29.8)	1	1
Interview	Low strain, isolated	945 (22)	**1.20 (1.10**–**1.31)**	**1.24 (1.14**–**1.36)**
1991–1995	Passive, collective	437 (10.2)	1.05 (0.94–1.17)	**1.18 (1.05**–**1.33)**
	Passive, isolated	518 (12.1)	**1.59 (1.43**–**1.77)**	**1.57 (1.41**–**1.75)**
Sickness absence	Active, collective	283 (6.6)	**1.64 (1.44**–**1.87)**	**1.66 (1.45**–**1.90)**
1993–1998	Active, isolated	331 (7.7)	**1.61 (1.42**–**1.83)**	**1.64 (1.44**–**1.86)**
	High-strain, collective	202 (4.7)	**1.59 (1.37**–**1.86)**	**1.83 (1.57**–**2.13)**
	High-strain, isolated	294 (6.9)	**1.61 (1.41**–**1.84)**	**1.69 (1.48**–**1.93)**
				
**Period 2**	Low strain, collective	909 (23)	1	1
Interview	Low strain, isolated	821 (20.8)	0.94 (0.85–1.03)	**0.90 (0.82**–**1.00)**
1997–2001	Passive, collective	358 (9.1)	1.10 (0.97–1.24)	1.03 (0.91–1.17)
	Passive, isolated	462 (11.7)	1.09 (0.98–1.23)	1.09 (0.97–1.22)
Sickness absence	Active, collective	336 (8.5)	**1.31 (1.15**–**1.49)**	**1.27 (1.11**–**1.44)**
1998–2004	Active, isolated	430 (10.9)	**1.44 (1.28**–**1.62)**	**1.44 (1.27**–**1.62)**
	High-strain, collective	232 (5.9)	**1.39 (1.20**–**1.61)**	**1.28 (1.10**–**1.49)**
	High-strain, isolated	402 (10.2)	**1.74 (1.55**–**1.96)**	**1.82 (1.61**–**2.06)**
				
**Period 3**	Low strain, collective	1081 (23.6)	1	1
Interview	Low strain, isolated	1026 (22.4)	**0.90 (0.82**–**0.99)**	**0.92 (0.84**–**1.00)**
2003–2007	Passive, collective	400 (8.7)	**1.16 (1.03**–**1.31)**	**1.17 (1.04**–**1.32)**
	Passive, isolated	559 (12.2)	**1.23 (1.11**–**1.37)**	**1.33 (1.19**–**1.48)**
Sickness absence	Active, collective	313 (6.8)	**1.22 (1.07**–**1.39)**	**1.34 (1.17**–**1.53)**
2004–2010	Active, isolated	459 (10)	**1.43 (1.27**–**1.60)**	**1.57 (1.40**–**1.77)**
	High-strain, collective	264 (5.8)	**2.01 (1.75**–**2.30)**	**2.28 (1.98**–**2.61)**
	High-strain, isolated	473 (10.3)	**2.15 (1.92**–**2.40)**	**2.38 (2.13**–**2.67)**
				
**Period 4**	Low strain, collective	722 (21.7)	1	1
Interview	Low strain, isolated	671 (20.2)	1.06 (0.95–1.19)	1.05 (0.94–1.17)
2009–2013	Passive, collective	271 (8.2)	**1.17 (1.01**–**1.35)**	1.13 (0.97–1.30)
	Passive, isolated	375 (11.3)	0.99 (0.87–1.13)	0.96 (0.84–1.09)
Sickness absence	Active, collective	227 (6.8)	1.09 (0.93–1.28)	1.13 (0.97–1.33)
2010–2016	Active, isolated	370 (11.1)	1.12 (0.98–1.27)	**1.18 (1.03**–**1.35)**
	High-strain, collective	226 (6.8)	**1.38 (1.18**–**1.61)**	**1.32 (1.13**–**1.54)**
	High-strain, isolated	459 (13.8)	**1.92 (1.70**–**2.16)**	**1.97 (1.75**–**2.23)**

a Adjusted for age and educational level.

The iso-strain profile had the highest IRR level in periods 2, 3, and 4 and second-highest during period 1. During period 1, all profiles had statistically significant increases compared with the reference group. During the turbulent period 2, only the high-demand profiles were statistically elevated. During period 3, all profiles except the low strain isolated profile had elevated levels, and finally, during period 4, none of the 4 low demands profiles had statistically significant elevated levels.

## 4 Summary and comments

The first aim of the present study was to investigate changes over time (1991–2013) in exposure profile groups characterised by combinations of low or high levels of job demands, job control, and social support. The main finding was the increase in exposure to high-strain work (high job demands, low job control) among both care workers and in the all-other occupations group.

A second aim was to investigate the relations between the various exposure profiles and compensated sickness absence days for the three years following the interview. The main finding was, that, regardless of occupational group, the iso-strain profile together with the high-strain collective profile were characterised by higher levels of absence due to sickness before as well as during and after the millennium crisis. These findings on the number of sickness absence days were supported by a corresponding pattern with a high share of workers with compensated sickness absence for more than 14 days in these profiles.

Another finding was that the mentioned pattern for absence days remained also when differences between exposure profiles were tested by Incident Rate Ratios with pooled data to four periods and adjustments for age and education level. In the adjusted model, almost all four high-demand profiles had statistically significant elevated levels during all four periods. A final finding was that the increase in the number of sick days around the millennium crisis was consistently much higher among care workers than among employees in all other occupations groups. During the 23 years covered by the study (1993–2016), the care workers, with a few, minor exceptions had a higher average of compensated days on sickness absence than the other occupations.

In the present study, we included social support, which is an additional dimension to what had been applied in the time series published biannually by Statistics Sweden (Swedish Work Environment Agency, 2014). The completion of social support in our study proves to have some explanatory value (Table 3). In general, other aspects being equal, the sickness absence pattern over the years shows that high social support was related to somewhat lower sickness absence. Recent research has shown that high social support may have a paradoxical effect on health and sickness absence (Aronsson et al., 2021). Social support is supposed to lower sickness absence through its health effects but may also raise the motivation to go to work when ill, which in the long run may harm the health and raise sickness absence. Further studies should consider this paradox.

### 4.1 Changes in the proportions of workers in the different exposure profile groups

We have tried to relate and interpret the exposure changes to changes on the organisational and the labour market level. Since we lack systematic individual and aggregated data for statistical-based correlation analyses, assumptions must rely on ecological observations that can serve as a basis for further mediation and multi-level research.

Among care workers, the iso-strain profile was the one which increased the most, as its proportion more than doubled from 1993 to 2003. The steepest upturn in this profile occurred in the mid and late 1990s, which coincides with the time when there were increases in staff redundancies and sickness absence. During this period, the active isolated profile increased sharply and at the same time, there was a sharp increase in sickness absence. The largest proportional reduction can be observed for the low strain, collective profile. It is interesting that none of these profiles later returned to the proportions they had before the turbulence during the late 1990s. For the all-other occupations group, the changes in exposure profile affiliation are in the same direction but much weaker than among the care workers. An interpretation is that the observed exposure changes, as they are indicated through the proportion of workers in the different profiles, reflect business cycles as well as continuous long-term structural changes.

Although the assessment of demands and control in the model we used is based on the assumption that the measured exposures reflect how to work is organised rather than workers’ reactions (Burr et al., 2016; Karasek et al., 1998; Karasek & Theorell, 1990; Theorell & Hasselhorn, 2005) – personal dispositions, mood, expectations, previous experiences or health have probably also influenced the ratings (Rugulies, 2012).

The observation period for our study was more than two decades, which raises the question of whether such personal factors may have changed over time among our participants in a way that makes comparisons over time scientifically questionable. Also, the amount and tone in which work stress is discussed in the media have probably changed over the observation period, which may have influenced the ratings.

### 4.2 Exposure profiles, turbulence, and compensated sickness absence among care workers

First, some words about the care worker sample from 1993 and their sickness absence 1994–96. One profile –high-strain collective – showed a deviant pattern in those years. One probable reason may be statistical as the total care worker sample was smaller in the 1993 sample and the high-strain collective profile was the smallest numerically. In line with what could be expected, the highest rates of sickness absence among care workers were found for the iso-strain profile. The lowest sickness absence and the smallest variation in sickness absence were found among the passive profiles, which is somewhat unexpected. However, a few studies have focused on these profiles (Mutambudzi et al., 2019; Suominen et al., 2007), thus it is difficult to find a consistent pattern over studies.

The peak level of sickness absence in the passive profiles was considerably lower than in the high demands profiles, but the percentage increase was still considerable, with a doubling or more of the number of days around 2000. When sickness absence peaked around the year 2000, the passive jobs were more favourable in terms of sickness absence. During less volatile times, such as after 2008, the difference in sickness absence between the high demands and low demands profiles was small. During the period 2009–2013 (sickness absence 2010–16), when sickness absence increased again in Sweden the iso-strain profile has the highest IRR followed by two other high-strain profiles.

Previous research has shown that meso-level changes in the form of staff reductions and reorganisations have an impact on job strain and health through changes in the exposure structure, for example, in the balance between job demands and control for the individual worker (Korunka et al., 2003; Vahtera & Kivimaki, 2009; Westerlund et al., 2004). However, none of these studies has investigated sickness absence in relation to the types of job demands-control-support exposure profiles used in the present study. In the present study, workers in passive and low strain jobs had a comparatively low increase in sickness absence during the years of heavy downsizing in the late 1990s. A macro-level-oriented explanation for these somewhat unexpected results could be that passive and low strain jobs are less sensitive to the intensification of labour through macro-changes than jobs with high demands. In high-demand jobs, increases in external pressure may eliminate the rest of individual adjustment opportunities to high workload.

During the economic downturn and staff cuts in the private sector from 1991 to 1995, the increase in sickness absence was very small or zero. This can be explained by an economic and personnel decline in the private sector driven by reduced demand for the company's products, which can lower labour intensity. For staff who survive cuts in the public sector in the health and care professions, the result may be increased labour intensity as the need for health care and care is unchanged. This may be one component in the explanation of the increased absence due to illness and the differences between the care sector workers and the other occupations.

Finally, there was some overlap between the periods of ups and downs in sickness absence, i.e., the created three-year intervals are not pure ups and downs in general sickness absence in Sweden. There is no easy methodological solution to this problem, but hopefully, it evens out in the long run between years of high and low absenteeism.

### 4.3 Further research

Several studies in recent decades have discussed the changing nature of work and its determinants (Cooper, 2006, 2009). The present study contributes to this line of research by analysing the changes in job demands-control-social support exposures.

First, despite all of the attempts to create more sustainable working conditions, the proportion of people in so-called iso-strain jobs is growing, especially in health care work. More knowledge about the development and the mechanisms behind this trend would be highly relevant for the design of preventative measures.

Second, during a period of large staff cuts, it seems that those with high-strain jobs have small margins for extra workload and are more likely to face or experience increased sickness absence. For preventative efforts, more research is needed in order to understand the organisational and individual mechanisms behind this uneven distribution of labour intensification.

Third, there are indications that changes at macro and meso-levels are mediated to illness and to sickness absence by changes in psychosocial exposure. More research-based knowledge of these processes and the mediators are called for in a world with a globalised economy and a pandemic causing instability. Indeed, such knowledge will be essential when facing rapidly emerging economic recessions that may quickly propagate into budget deficits that lead to staff cuts and major consequences for health and sickness absence. This is what is happening now and will happen even more in the future in many countries in response to the corona pandemic. These research issues require collaboration between social epidemiologists and social scientists.

### 4.4 Strengths and weaknesses

This study was based on a very large collection of data including biennial (from 1991 to 2013) measures of work environment variables. The response rate for the Work Environment Surveys was high (66%–89% for the study period) (Swedish Work Environment Agency, 2014). Well-tested instruments with high psychometric quality were used (Wikman, 1991). A further strength is that work environment indicators have been measured independently of sickness absence figures, the latter of which was obtained from the Swedish Social Insurance Register (1993–2016) and thus has high precision.

An additional strength was that the register data used only applies to absence due to the worker's illness - i.e., not absence due to pregnancy, care of a sick child or care of other persons etc. To our knowledge, no other studies applying the job demands-control-social support model have analysed all registered sickness absences over a period as long as three years after the interview and exposure measurement.

One limitation is that the study lacks sickness absence data for periods shorter than two weeks, as such absence is compensated by the employer and is not registered in the Social Insurance Register. The extent to which this affects the results, and their generalisability is difficult to assess. Short absences may in some cases prevent longer sickness absences by allowing employees to recover before health problems potentially worsen. One hypothesis for further research could be that unregistered short-term absences are more frequent among employees in passive jobs, suggesting that factors characteristic of the passive profile could partly explain the lower sickness absence. Our comparison group, “all other occupations”, is comparatively larger and more heterogeneous. For purely statistical reasons, the variation in sickness absence rates is therefore smaller. Swings in different directions in different subgroups dampen the total variation, which is a reason for taking caution when making conclusions about differences.

The “ecological” findings of associations between factors at the macro and meso-contextual levels and job demands-control-social support structures and sickness absence are based on contemporaneity. More rigorous scientific methods are needed to investigate this in further research, especially finding links between macro, meso, and micro levels. A further limitation is that we cannot assess underlying health conditions among the participants, which to a great extent could affect the level of sickness absence.

## 5 Conclusions

The study draws attention to the development of health-threatening exposure profiles based on levels of job demands, control, and social support in relation to the investigated care occupations. The levels of demands for some exposure profiles appear to be at such a level that any increase in demands would be difficult to compensate for through more job control or more social support. This is especially true for the iso-strain profile but also for the active collective profile which is supposed to balance job demands and control and therefore should be an ideal and attractive profile.

A second conclusion is that during periods of increasing and generally high sickness absence, the contribution to the increase is different within the different exposure profiles. The increase is significantly greater, and the absolute levels are clearly higher in the high-strain and active profiles than in the passive profiles. This pattern is more pronounced within the two studied occupational groups in the health sector than within the comparison group all other occupations. In accordance with other studies, the consequences of economic downturn and staff redundancies need not be immediate but can be delayed. In relation to the severe pressure on the health and care sector due to the corona pandemic, this is a serious warning sign for the future.

## Declaration of interest

Declarations of interest: none.

## Ethical considerations

The regional ethical board in Stockholm approved this study (Dnr. 2018/223-31/5) and waived the requirement that informed consent from the research subjects should be collected.

## Data sharing statement

The data which this study builds upon are available from Statistics Sweden. The data are not publicly available but can be used if an ethics permission is received.

## Author statement

Gunnar Aronsson: Conceptualization, Roles/Writing - original draft, Writing - review & editing.

Constanze Leineweber: Conceptualization, Writing - review & editing.

Staffan Marklund: Conceptualization, Writing - review & editing.

Magnus Helgesson Conceptualization; Data curation; Formal analysis; Funding acquisition; Investigation; Methodology; Project administration; Resources;

Validation; Visualization; Roles/Writing - original draft; Writing - review & editing.

## Ethical statement

The regional ethical board in Stockholm approved this study (Dnr. 2018/223-31/5) and waived the requirement that informed consent from the research subjects should be collected. The Swedish law on Research Ethics states that the use of register data which has been given without consent and contains sensitive information (e.g., regarding health conditions) must get approval from a Regional Research Ethics Committee. This applied to our SA data. Participation in the Work Environment survey was based on informed content. The approval must be sought for research use of personal information also where anonymization has taken place after the data linkage.
